# NGMA-3. Use of multi-omics data to initialize and validate a causal model of glioblastoma stem cell signaling

**DOI:** 10.1093/noajnl/vdab070.018

**Published:** 2021-07-05

**Authors:** Emilee Holtzapple, Natasa Miskov-Zivanov, Brent Cochran

**Affiliations:** 1 Computational and Systems Biology, University of Pittsburgh, Pittsburgh, PA, USA; 2 Electrical and Computer Engineering, Bioengineering, Computational and Systems Biology, University of Pittsburgh, Pittsburgh, PA, USA; 3 Tufts University School of Medicine, Boston, MA, USA

## Abstract

Glioblastomas and glioblastoma stem cells are heterogeneous with respect to mutations, gene expression, and response to drugs. To make predictive responses of individual GBM stem cell lines to drugs, we have constructed a causal model of glioblastoma stem cell signaling. The core model was built starting from pathways identified from TCGA mutation data with the addition of the Jak/STAT, Hedgehog, and Notch pathways. Elements and relations between them were validated and extended using the PCNet interaction database and the INDRA database which includes machine read extractions from the biomedical literature. The result is a high confidence executable model consisting of 209 element and 370 rules of interaction between the elements. Stochastic simulations of the model provide dynamic (quantile) changes in time and responses to perturbations. The output provides activity of individual nodes as well as a cellular output state of cell cycle progression, apoptosis, or differentiation. To simulate the responses of individual cell lines to kinase inhibitors, the model was initialized using DNA sequencing data, RNA-seq, and reverse phase protein array (RPPA) data from each cell line. Comparing the results of the simulations to the drug responses of 11 different kinase targets, the model was 88% accurate in predicting effects on growth and survival. The model was further tested by comparing the effects of Mek inhibition of each of the cell lines in model to the results observed in the RPPA data which overlap by 127 elements. In this case, there was 62% concordance between the model and data when binned into quintiles. Discrepancies between the model predictions and the data are being investigated to determine whether the model logic or extent needs to be revised to improve the model. This modeling approach is a step toward developing algorithms for personalized therapeutics for GBM based on multi-omics data.

